# The Effect of Effervescent and Buffered Alendronate Compared to Conventional Alendronate on Markers of Bone Turnover: A Randomized Non-inferiority Trial

**DOI:** 10.1007/s00223-023-01140-w

**Published:** 2023-10-06

**Authors:** Rawan Hikmet, Torben Harsløf, Bente Lomholt Langdahl

**Affiliations:** 1 Department of Endocrinology and Internal Medicine, Aarhus University Hospital, Palle Juul-Jensens Boulevard 99, DK-8200 Aarhus N, Denmark; 2 Department of Clinical Medicine, Aarhus University, Aarhus, Denmark

**Keywords:** Osteoporosis, Alendronate, CTx, PINP

## Abstract

Buffered and effervescent alendronate (ALN-EFF) increases gastric pH and is reported to decrease the risk of gastrointestinal side effects compared to conventional formulations of alendronate (ALN). The clinical effectiveness of ALN-EFF, however, has not been investigated. This study aims to investigate if ALN-EFF is non-inferior to ALN in suppressing bone turnover markers (BTM). We conducted a 16-week prospective, randomized, open-label study comprising 64 postmenopausal women with BMD T-score < −1 naïve to osteoporosis treatment. Participants were randomized 1:1 to ALN or ALN-EFF. We collected blood samples at 0, 4, 8, and 16 weeks. Non-inferiority margin was determined as 12% (80% of efficacy retained), and an SD of 15% on change in CTx. CTx decreased by 58.2% ± 24.1% in the ALN group and by 46.9% ± 23.3% (CI − 38.42:− 55.35) in the ALN-EFF group (*p* = 0.08). The non-inferiority limit was 46.6%. With ALN-EFF the CI crosses the non-inferiority limit thus the test for non-inferiority was indeterminate. PINP decreased by 45.7 ± 22.6% in the ALN group and by 35.1 ± 20.7% in the ALN-EFF group (*p* = 0.07). Changes over time in the BTMs were not significantly different between the groups, *p* > 0.10 for both CTx and PINP. There was no difference in frequency of AEs or compliance between the two groups, but rate of discontinuation was lower with ALN-EFF. In conclusion, suppression of BTMs was not significantly different between the groups but formal non-inferiority could not be established.

## Introduction

Osteoporosis is a chronic disease characterized by increased skeletal fragility and risk of fractures. Alendronate is mainstay in the treatment of postmenopausal osteoporosis as it significantly reduces the risk of hip- and vertebral fractures. The effect is gained as early as 12–18 months after initiation of treatment [1]. Alendronate (ALN) is an acid that binds to the bone mineral hydroxyapatite, inhibits osteoclast function, reduces bone resorption and thereby increases bone mass and strength [2].

The level of bone turnover can be monitored by the bone turnover markers (BTM) C-terminal telopeptide type I collagen (CTx) that is released during bone resorption and collagen degradation and N-terminal procollagen type I propeptide (PINP) that is released during bone formation and collagen formation. With alendronate treatment bone turnover is maximally suppressed within 12–14 weeks [3]. Although alendronate is cheap and effective, compliance is low. This is mainly due to gastrointestinal (GI) side effects [4] which are believed to be caused by the low gastric pH associated with alendronate ingestion [5]. With a buffered and effervescent solution of alendronate (ALN-EFF), however, no retention of the medication is seen in the oesophagus and gastric pH increases [6]. In accordance with this, uncontrolled studies with ALN-EFF showed fewer GI side effects than what has been reported for conventional alendronate [7] as well as fewer patients discontinuing therapy [8]. The effect of the effervescent formulation on clinical parameters such bone turnover, bone mineral density, or fracture risk, however, has not been investigated. We therefore conducted a clinical trial to investigate if the effect of ALN-EFF on BTM is non-inferior to the effect of conventional ALN.

## Methods

### Study Design

We conducted a 16-week prospective, randomized, open-label study comprising 64 postmenopausal women with a BMD T-score <− 1. Participants were randomized 1:1 to two groups of 32 women each. One group received branded ALN and the other ALN-EFF 70 mg weekly. In addition, all women received daily supplementation of 800 mg calcium and 38 µg vitamin D.

We conducted the trial according to the Helsinki Declaration, the Danish Health Law, and GCP guidelines. The Danish Data Protection Agency (1-16-02-306-21), The Regional Ethics Committee (Eudract No. 2020-005040-35) and The Danish Health and Medicine Authority (2020-120512) approved the study. The local Good Clinical Practice (GCP) at Aalborg and Aarhus University Hospital monitored the trial. The trial is registered at clinicaltrials.gov (identifier NCT05325515).

### Study Participants

We included postmenopausal women (2 years since last menstrual bleeding) with a BMD T-score <−1. Moreover, as the primary endpoint was decrease in CTx (see below), we included women with CTx > 420 ng/L which is the median value for postmenopausal women at our laboratory. We excluded participants who had ever received treatment for osteoporosis, had contraindications for alendronate (according to the SPC), fulfilled the criteria for teriparatide treatment in Denmark, received treatment with systemic glucocorticoids within the past 12 months, received hormone replacement therapy, had rheumatoid arthritis, inflammatory bowel disease, untreated thyroid disease, primary hyperparathyroidism, diabetes mellitus, unstable liver disease, cancer within the past 2 years (except basal cell carcinoma of the skin), major GI disease within the past 12 months, estimated glomerular filtration rate (eGFR) < 60 mL/min, or p-25-hydroxy-vitamin D (D3 + D2) < 50 nmol/L.

### Randomization

We randomized the participants to one of the two treatment arms by 1:1 randomization. We did the randomization in blocks of 8 and randomization was performed using REDCap [9]. The randomization algorithm was generated by the local REDCap support. Study data were collected and managed using REDCap electronic data capture tools hosted at Aarhus University, Denmark.

### Procedures

We recruited participants via the dual-energy x-ray absorptiometry (DXA) service at the Department of Endocrinology and Internal Medicine, Aarhus University Hospital, Denmark. All participants were referred from their general practitioner or other hospital department for evaluation by DXA. We identified women with osteopenia or osteoporosis (BMD T-score <− 1 at lumbar spine or total hip) and reviewed their medical records for any exclusion criteria. Women who were still eligible at this stage were sent a letter with written information about the project and contacted the investigator by telephone if they were interested in further information and participation in a screening visit.Written informed consent was obtained from all participants included in this study before the first procedures were performed.

At the screening visit, we acquired information on medical history, prescription medication, calcium intake, age at menopause, smoking history and performed a medical examination. Furthermore, a screening blood test was analyzed to rule out presence of the exclusion criteria mentioned above. Bone turnover marker CTx was measured and women with CTx > 420 ng/L were included if they did not meet any exclusion criteria.

**Fig. 1:**
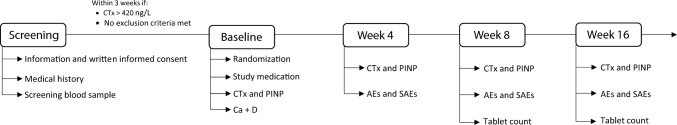
Study design

We collected fasting blood samples at baseline and at weeks 4, 8, and 16. Information on adverse events (AEs) was obtained at each visit. Participants were provided with tablets for the entire study period at baseline and were instructed regarding the administration of the medication. The ALN group should swallow the pill with a glass of water and the ALN-EFF group had to dissolve the effervescent tablet in water and drink it immediately. Both groups were instructed to take the medication fasting in the morning and allow 30 min before consuming food/drinks and that they should not lay down but remain upright. At week 8 and week 16 participants were instructed to bring the remaining tablets and compliance was assessed by tablet count (Fig. 1). Furthermore, they were provided with Unikalk Forte and were instructed to take one tablet twice a day.

### Biochemistry

Blood samples were collected between 7.30 and 10.00 am following an overnight fast. Samples were then left to clot for 30 min and afterwards centrifuged. The serum and plasma were stored at − 80 °C until analysis at the end of trial. BTM were analyzed using EDTA plasma. All BTMs were analyzed in one single batch to reduce variation in the analysis. We measured BTMs using an electrochemiluminescent immunoassay on a Roche COBAS 8000 reader (Roche, Rotkreuz, Switzerland). At our facility the least significant change (LSC) for PINP is 22.9% and for CTx 33.0% and the coefficiency of variation is 10% for CTx and 7.4% for PINP.

### Outcomes

Our primary endpoint was percent change in CTx from baseline to end of the study (after 16 weeks). The secondary endpoints were percent change in PINP from baseline to end of study (EOS), rate of decline of CTx and PINP, compliance and adherence to the treatments, AEs, and serious adverse events (SAEs).

### Statistical Analysis

The sample size was determined based on the primary endpoint. With a minimum expected decrease in CTx of 60% with ALN, a non-inferiority margin of 12% (80% of efficacy retained), an SD of 15% on change in CTx, and a significance level of 5%, 54 participants should be included in the study in order to obtain statistical power of 90%. We adjusted this to 64 participants (32 in each group) to account for dropouts. Baseline variables were checked for normality using qq-plots and log-transformed as appropriate. Data are displayed as means ± SD for normally distributed data and median with CIs for non-normally distributed data. We calculated percent changes in BTMs from baseline to EOS, checked normality of data using qq-plots and compared differences between groups using independent samples *t* test. For the calculation of change in BTMs we used the value from the baseline visit to minimize variation. We furthermore assessed differences in changes in BTMs over time using a general linear model with repeated measures. Differences in AEs and SAEs in the two treatment groups were assessed using non-parametric statistics.

## Results

### Baseline Characteristics

**Fig. 2:**
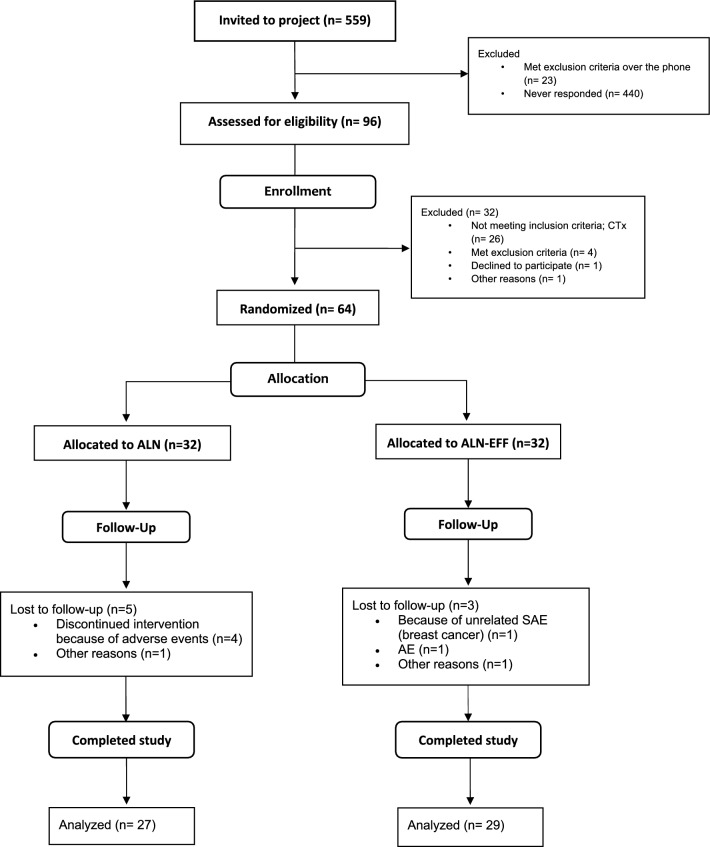
Flow of participants through the study from invitation to analysis

A total of 559 women were invited to the study, of whom 440 did not respond. Twenty-three were excluded after an interview by phone due to the presence of exclusion criteria. Ninety-six patients were screened from September 27, 2021, to February 07, 2022, and 64 participants were included in the trial. Thirty-two were excluded (26 patients did not meet the inclusion criteria CTx > 420 ng/L and 4 patients met one or more exclusion criteria), one patient withdrew informed consent, and one patient was not included as the number of participants needed in the study had been reached. Of the 64 participants, 56 completed follow-up at 16 weeks (Fig. 2).

Table 1 shows that baseline characteristics were well balanced between the two groups. Mean age was 61.9 ± 6.0 years and 61.8 ± 6.2 years, height was 166.8 ± 4.5 cm and 165.4 ± 7.3 cm, weight was 68.0 ± 11.0 kg and 65.0 ± 11.9 kg, and all women were post-menopausal with a mean of 11.9 ± 5.7 years and 14.0 ± 6.2 years since menopause in the ALN-EFF group and ALN group, respectively. All participants had osteopenia or osteoporosis. The mean *T*-score of the spine was − 1.9 ± 0.8 and − 1.7 ± 0.6 and mean T-score of total hip was − 1.5 ± 0.6 and − 1.4 ± 0.6, respectively. Biochemistry, number of previous fractures, alcohol consumption, and smoking status were similar between the two groups.

**Table 1:** Baseline characteristics

Study population (*n* = 64)	ALN-EFF (*n* = 32)	ALN (*n* = 32)
Age (years)	61.9 ± 6	61.8 ± 6.2
Height (cm)	166.8 ± 4.5	165.4 ± 7.3
Weight (kg)	68.0 ± 11.0	65.0 ± 11.9
BMI	24.1 ± 3.3	24.0 ± 4.1
Years since menopause	11.9 ± 5.7	14 ± 6.2
Number of participants with osteoporosis (*T*-score ≤ −2.5)	6	5
*T*-score		
Lumbar spine (L_1_–L_4_)	− 1.9 ± 0.8	− 1.7 ± 0.6
Total hip	− 1.5 ± 0.6	− 1.4 ± 0.6
Femoral neck	− 1.8 ± 0.6	− 1.8 ± 0.5
Bone mineral density (g/cm^2^)		
Lumbar spine (L_1_-L_4_)	0.836 ± 0.089	0.855 ± 0.066
Total hip	0.754 ± 0.078	0.766 ± 0.075
Femoral neck	0.751 ± 0.079	0.769 ± 0.073
Biochemistry		
CTx (ng/L)	610 (560, 660)	630 (570, 690)
PINP (µg/L)	72.33 (63.26, 74.34)	77.85 (69.91, 84.23)
TSH (mIU/L)	1.4 (1.2, 1.7)	1.5 (1.3, 1.9)
PTH (pmol/L)	4.8 (4.3, 5.3)	5.2 (4.7, 5.7)
eGFR (mL/min/1.73 m^2^)	83.2 (80.1, 86.5)	83.2 (80.3, 86.2)
25-OH vitamin D (nmol/L)	101 ± 26	96 ± 23
Creatinine (µmol/L)	64.5 ± 9.9	64.6 ± 7.8
Ionised Ca^2+^(mmol/L)	1.25 ± 0.03	1.24 ± 0.03
Any previous fracture		
Yes	13	15
No	19	17
Alcohol consumption, weekly		
< 7 units	28	26
7–14 units	4	6
> 14 units	0	0
Smoking status		
Current	2	2
Previous	15	14
Never	15	16

Biochemical results displayed are from the screening visit. Data are shown as mean ± SD except for CTx, PINP, TSH, PTH and eGFR which are shown as median (95% CI)

^a^eGFR measurements at the laboratory doesn’t exceed 90 mL/min/1.73 m^2^, therefore all measurements of eGFR > 90 are stated as 90 mL/min/1.73 m^2^

*ALN* alendronate, *ALN-EFF* effervescent and buffered alendronate, *BMI* body mass index, *CTx* collagen I cross-lined C-terminal telopeptide, *PINP*: procollagen type I N-terminal propeptide, *TSH* thyroid-stimulating hormone, *PTH* parathyroid hormone, *eGFR* estimated glomerular filtration rate

### Change in BTMs

**Fig. 3:**
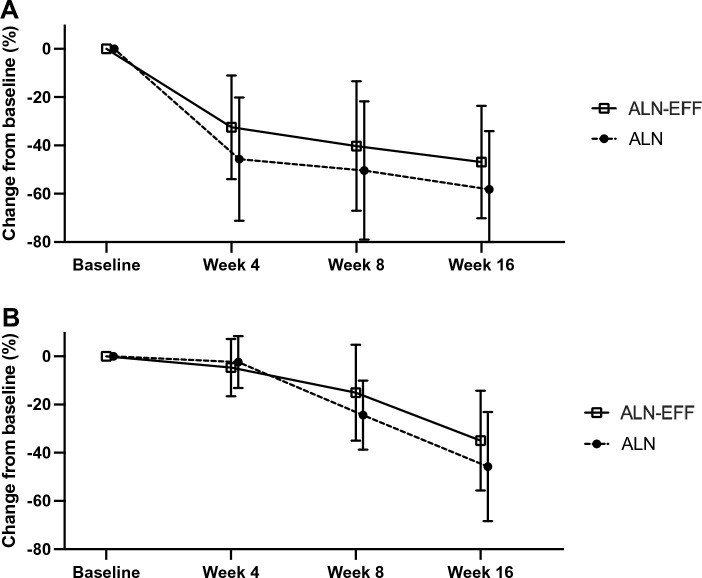
Percentage change in BTMs. Mean percent change from baseline with standard deviation for **A** bone resorption marker (CTx) and **B** bone formation marker (PINP). The two groups are intentionally displaced from each other for a better visualization of data. *ALN-EFF* effervescent and buffered alendronate, *ALN* branded alendronate

From baseline to EOS CTx in the ALN group decreased by 58.2%. As we wanted to retain 80% of this effect with ALN-EFF the non-inferiority limit was 46.6%. With ALN-EFF CTx decreased by 46.9% (CI − 38.42: − 55.35) and as the CI crosses the non-inferiority limit the test for non-inferiority is indeterminate. CTx and PINP decreased in both treatment groups (Table 2). Comparing the changes in the two groups using independent samples t test there was no significant differences as CTx decreased by 58.2 ± 24.1% in the ALN group and by 46.9 ± 23.3% in the ALN-EFF group (*p* = 0.08) and PINP decreased by 45.7 ± 22.6% in the ALN group and by 35.1 ± 20.7 in the ALN-EFF group (*p* = 0.07). Looking at individual changes, 20 participants (74%) in the ALN group and 22 participants (76%) in the ALN-EFF group had a decrease in CTx below LSC (data not shown, *p* = 0.36) and for PINP 22 participants in both groups had a decrease beyond LSC (data not shown, *p* = 0.75). Using a general linear model with repeated measures there was no interaction between treatment group and time for change in CTx or PINP (*p* > 0.10 for both) meaning that the changes over time in the BTMs were not significantly different between the groups (Fig. 3).

**Table 2:** Percent change from baseline (week 0) to week 16 in CTx and PINP for the two treatment groups (ALN-EFF and ALN)

	ALN-EFF (*n* = 29)	ALN (*n* = 27)	*p* value
CTx	− 47 ± 23	− 58 ± 24	0.08
PINP	− 35 ± 21	− 46 ± 23	0.07

Differences in changes from baseline to end of study were compared using independent t test. Shown as mean ± SD

*ALN-EFF* effervescent and buffered alendronate, *ALN* alendronate, *CTx* collagen I cross-lined C-terminal telopeptide, *PINP* N-terminal procollagen type I propeptide

### Adverse Events

The total number of reported AEs were 49 in the ALN-EFF group and 41 in the ALN group (Table 3). Regarding gastrointestinal symptoms there was no overall difference between the groups (*p* = 0.6). Reflux was reported two times in the ALN-EFF group and five times in the ALN group. Diarrhea/obstipation were more frequent for participants receiving ALN-EFF with nine women reporting this AE and five in the ALN group. Gastrointestinal pain and nausea were reported equally between the groups and reduced appetite only in the ALN-EFF group (two participants). There was no difference in duration of gastrointestinal symptoms (data not shown). Musculoskeletal symptoms appeared more frequently in the ALN group with five reported AE compared to two in the ALN-EFF group. Furthermore, the reported duration of these symptoms was less than a week for every individual in the ALN-EFF group while five subjects in the ALN group reported symptoms lasting over 1 month (data not shown). Dryness in mouth and sensitive gingiva appeared more frequent with five cases in the ALN-EFF group and two in the ALN group. Fever, and headache were reported equally between the two groups. Finally, there was one SAE in the ALN-EFF group and none in the ALN group. The SAE (breast cancer) was not related to this study or the study drug. Four participants in the ALN group and one in the ALN-EFF group discontinued the study because of AEs. The cause of discontinuation in the ALN group was reflux, abdominal pain, obstipation, and eye symptoms and in the ALN-EFF group, one participant had frequent migraines.

**Table 3:** Adverse events

Adverse event, *n* (%)	ALN-EFF (*n* = 32)	ALN (*n* = 32)	*p* value^c^
Any gastrointestinal symptoms	22 (69)	19 (59)	0.6
Reflux	2 (6)	5 (16)	
Gastrointestinal pain	5 (16)	6 (19)	
Diarrhea/obstipation	9 (28)	5 (16)	
Nausea	4 (13)	3 (9)	
Reduced appetite	2 (6)	0 (0)	
Musculoskeletal symptoms^a^	2 (6)	5 (16)	0.43
Headache	2 (6)	3 (9)	> 0.99
Fracture	1 (3)	1 (3)	> 0.99
Dryness in mouth/sensitive gingiva	5 (16)	2 (6)	0.43
Infection/fever (unspecified)	5 (16)	4 (13)	> 0.99
SAE (breast cancer)	1 (3)	0 (0)	> 0.99
Other^b^	12 (38)	7 (22)	0.27
Event leading to discontinuation of participation	1 (3)	4 (13)	0.35

*ALN-EFF* effervescent and buffered alendronate, *ALN* alendronate, *SAE* serious adverse events

^a^Bone pain and arthralgia

^b^Other adverse events reported: eye pain, chest pain, nail changes, hair loss, sleep problems, eczema, jaw pain, edema, bloated stomach, lower back pain, neck pain, pain in ribs, cramp in the feet, stinging sensation in face

^c^Groups were compared using Fisher’s exact test

### Compliance

More than 80% of the expected drug intake at week 16 was defined as good compliance with the medication. The compliance was good in both groups among participants who completed the 16-week trial, as 100% in the ALN-EFF group and 96.3% in the ALN group were compliant, respectively. One participant in the ALN group had a compliance of 75%.

## Discussion

In the present study we investigated the effect of effervescent and buffered alendronate compared to conventional alendronate on suppression of BTMs in a randomized non-inferiority study in postmenopausal women with low bone density. The decrease in BTMs were not significantly different between the two groups but the test for non-inferiority for ALN-EFF was indeterminate as the CI for suppression in CTx crossed the non-inferiority limit. This means that based on the study design (non-inferiority trial) and the non-inferiority limit selected it cannot be determined whether the effervescent formulation is able to suppress bone turnover to the same extent as the conventional formulation.

Alendronate is the most widely used treatment for osteoporosis but is often discontinued due to GI side effects. Alternative treatments with comparable anti-fracture efficacy often require parenteral administration and therefore oral alternatives are warranted and one such alternative could be effervescent and buffered alendronate. The present study is the first to study the effect of ALN-EFF on a clinical parameter albeit only on BTMs. Compared to ALN the effect on BTMs from baseline to EOS was not statistically different nor was the rate of change during the study period. This suggests that the suppression of bone turnover with the two formulations may be comparable but this cannot be formally concluded. Whether effects are comparable or not regarding changes in BMD and fracture risk reduction also remains to be determined and would require a larger and longer study. One reason that the test for non-inferiority is indeterminate may be the variation in the CTx measurements. In a previous study, the SD on 12-week change in CTx was 12% [3]. For our sample size calculation, we increased the estimated SD to 15% to increase power but the SD turned out to be 23% in the ALN-EFF group and 24% in the ALN group. We have subsequently investigated possible analytical errors. According to the manufacturer hemolysis and biotin interference may affect the measurements but we have found no evidence of hemolysis in our samples and none of the study participants took dietary supplements containing biotin (data not shown). In addition, we reanalyzed ten randomly selected samples but got the same results (data not shown). Therefore, our results seem to be valid. We have, however, used a different assay for BTM than in the previous study where an IDS-iSYS automated immunoassays (Immunodiagnostic Systems, Boldon, UK) was used.

We also examined differences in AEs and SAEs between the two formulations. In general, there were no statistically significant differences between the two groups but this was also expected due to the sample size. In particular, the incidence of GI side effects was numerically similar between the two groups whereas side effects leading to discontinuation appeared fewer in those receiving ALN-EFF. To date two published clinical studies have examine side effects with ALN-EFF. In one observational cohort the one-year incidence of upper GI AEs was approximately 10% but there was no control group in the study [7]. Another study examined persistence with ALN-EFF in comparison to ALN as well as discontinuation due to GI AEs and found ALN-EFF to be superior in both respects [10] but the control group was a historical cohort of ALN users and the study therefore not randomized. Therefore, well-designed studies powered to demonstrate differences in AEs remain to be performed.

Strengths of the present study include its randomized design and choice of relevant comparator and study population. Limitations are the open-label design that may affect reporting of AEs. The study was not designed to investigate differences in side effects between the two groups as the relatively small sample size does not allow this. A further limitation is the duration that precludes analysis on harder endpoints as BMD and fracture risk. Moreover, we did not provide monitoring bottle caps on the pill glasses and therefore do not know if the medicine was ingested regularly.

In conclusion, we have investigated the effect of ALN-EFF compared with ALN on bone turnover and AEs in a randomized non-inferiority trial. Suppression of bone turnover was not significantly different between the groups but formal non-inferiority could not be established. The incidence of AEs did not differ between the two groups although fewer participants in the ALN-EFF group discontinued the study due to side effects.
